# Microbial Biofilms: From Molecular Mechanisms and Structure to Antimicrobial Therapy

**DOI:** 10.3390/microorganisms10081638

**Published:** 2022-08-12

**Authors:** Paolo Landini, Jaione Valle

**Affiliations:** 1 Department of Biosciences, University of Milan, 20133 Milan, Italy; 2 Instituto de Agrobiotecnología (Idab), CSIC-Gobierno de Navarra (CSIC-GN), 31192 Navarra, Spain

The first published observations that microorganisms associate to form microbial communities structured as biofilms in natural environments date back to the first half of the last century [1]. However, despite such early recognition of the ecological importance of microbial biofilms, their study was largely ignored in the following decades, as microbiologists focused mainly on microbial genetics and physiology, which are typically studied using pure cultures growing in a planktonic (i.e., single cell) state. It was only much later that research interest in microbial biofilms was rekindled, especially following the understanding that pathogenic bacteria growing in biofilms showed decreased sensitivity to antibiotics, thus leading to a higher risk of antimicrobial therapy failure [2]. Interest in biofilm studies has resulted in a dramatic acceleration of the research papers published in the field; indeed, according to the PubMed search engine (https://pubmed.ncbi.nlm.nih.gov/, accessed on 12 August 2022), the number of research articles featuring “biofilm” in their title went from 232 in the years 1981–1990 to 8258 in the year 2021 alone. The applications of genetic, and later genomic, metagenomics and transcriptomic techniques to biofilm studies, begun by work on *Escherichia coli* surface adhesion factors [3], has greatly advanced our knowledge of microbial biofilm biology, providing a much clearer picture of the differences in gene expression between planktonic and biofilm cells, of the phenotypic, biochemical and morphological properties of the bacterial cell [4], of the signal molecules involved in this process [5], and of how biofilm growth is associated with processes such as horizontal gene transfer and antibiotic resistance [6].

As mentioned above, biofilm growth represents a major concern in microbial infections, since it can lead to tolerance to antimicrobial treatments. This issue is directly connected to the growing emergence of antibiotic resistance as well as to the lack of novel antimicrobial agents. These are issues that, if not addressed, might lead to an estimated staggering amount of 10 million deaths/year caused by pan-antibiotic resistant pathogens by 2050 [7]. Lack of discovery of novel antibiotics has led to the exploration of alternative approaches for the development of antimicrobial therapies, including anti-virulence drugs, namely, molecules that can target virulence factors rather than cell growth. Biofilm formation is considered an essential target in such an approach, as biofilm inhibition would increase antibiotic effectiveness and make pathogens more sensitive to the host immune response. Therefore, novel therapeutic approaches focus on preventing the synthesis or disrupting the biofilm matrix components, thus rendering bacteria more sensitive to antibiotic treatments.

This Special Issue, *Microbial Biofilms: From Molecular Mechanisms and Structure to Antimicrobial Therapy*, includes research articles tackling crucial issues in the field of microbial biofilms. One paper published in this Special Issue points to the relevance of the pyrimidine biosynthetic gene *pyrD* as suitable target for anti-virulence and anti-biofilm agents in adherent invasive *Escherichia coli* (AIEC), an intracellular pathogen implicated in the onset of Crohn’s disease [8]. Another paper proposes a strategy for biofilm inhibition comprising the combinatorial analysis of chemical composition of essential oils from officinal plants and their effects on biofilm formation in *Pseudomonas aeruginosa* isolates from cystic fibrosis patients, using a machine learning algorithm aimed at the understanding of those molecules that are more active against biofilm formation [9]. Another interesting approach tackled in this Special Issue is the use of probiotic bacteria as therapeutic agents to inhibit biofilm formation by pathogenic microorganisms, either via the production of diffusible molecules or through direct competition. *Lactiplantibacillus plantarum* CCFM8724 appears promising in this respect, being able to impair the formation of a *Streptococcus mutans-Candida albicans* mixed-species biofilm, with supernatants of *L. plantarum* cultures strongly affecting the expression of *C. albicans* adhesion and virulence genes [10]. Other works in this Special Issue focus on biofilm formation by Staphylococci—the production of PIA (Polysaccharide intercellular adhesin), also called PNAG (β-1,6 poly-N-acetyl-glucosamine), is a main feature in these bacteria; however, an experimental polymorphism survey performed on *Staphylococcus aureus* clinical isolates from prosthetic joint-associated infections showed the absence of mutations affecting expression of the *icaABCD* operon, encoding PIA/PNAG biosynthetic genes. Single aminoacid changes in the IcaR regulatory protein resulting in increased PIA/PNAG production led to decreased virulence in a *Galleria mellonella* model, suggesting that biofilm formation might be detrimental to *S. aureus* virulence [11]. Surprisingly, a negative correlation was also observed between biofilm formation and resistance to some antibiotics in *S. epidermidis* clinical isolates, with no apparent role of PIA/PNAG in this process [12]. Thus, these two studies would suggest that, in Staphylococci, a possible trade-off exists between biofilm proficiency on the one hand and both virulence and antibiotic resistance on the other.

Altogether, the papers featured in this Special Issue cover some of the main topics in biofilm studies, mainly focusing on biofilm control. We believe these studies represent an important contribution to our knowledge in this field.
